# A comparison of data sources and models to project HRH needs in India: lessons from the universal health coverage planning process

**DOI:** 10.1186/1753-6561-6-S5-P11

**Published:** 2012-09-28

**Authors:** Devaki Nambiar

**Affiliations:** 1 Public Health Foundation of India, New Delhi, India

## Introduction

The path to universal health care in India requires concurrently addressing several human resource issues and is thus an opportunity to bring about much needed reforms in the health workforce. A key component of these reforms is systemic knowledge generation around what human resources for health (HRH) capacity currently exists in the country and what future needs may be projected to achieve universal health coverage. A lack of uniform sources of data has resulted in the use of diverse sources, which presents its own challenges in the planning process.

## Methods

Based on a qualitative comparative framework, we examine the implications of multiple data sources and models to estimate human resource needs for India over the next 30 years.

One set of comparisons projects estimates from census and sample survey data to adjusted figures from professional health councils to actually determine what the population coverage of various HRH cadres is (doctors, nurses and ANMS). Here we compare the data sources, assumptions and methodologies of HRH estimation approaches.

Another set of comparisons is of models that propose to achieve international norms of doctor-population coverage and nurse/midwife to doctor ratios, as compared to models that operate on more general health worker densities incorporating the role and contribution of community health workers and mid-level health practitioners. Here we compare theoretical bases, historical contexts and international evidence that justify various model and assess their relevance to the Indian case.

## Results

The use of different data sources results in estimation exercises with varying baselines and ultimately projects widely divergent human resources numbers and requirements. Major reliability and validity concerns may be raised with regard to typical sources of HRH data in India (the Census, the Medical Council of India, Indian Nursing Council, Sample Registration System). This is also related to a lack of inter-operability across these sources in terms of definitions and frequency in generation of health information.

Profound epistemological questions regarding India’s vision for health emerge in comparing doctor-driven models to community health worker-driven models or models that seek a middle path of mid-level practitioners. India’s successes with producing doctors has to be reconciled with the lack of a supportive environment for practice, its world renowned record of community health worker programmes has to be reconciled with the scale of coverage and resultant quality and training implications; finally, operationalizing mid-level practitioners across the country will require overcoming legal and normative barriers that privilege medical dominance and circumscribe what a legitimacy of HRH cadres.

## Discussion

Human resources projection exercises and sensitivity analyses raise key questions around the level of clinical competency across cadres, the functional implications of proposed new cadres, the paucity of “live” data, and epistemic considerations related to India’s vision for how to (human) resource universal health coverage.

Our recommendations include the use of human resources information management systems as part of management reform. There is an urgent need for systematic data generation related to HRH at appropriate levels in the country, supplemented by routinised cadre review. The country also needs to evolve uniform standards for regulation and accreditation, with appropriate (legal) attention to the scope and integration of practice across cadres at various levels of care. These structural changes require a careful balance between central guidance and strategic planning at the state level – in striking that balance, knowledge generation and management around HRH could improve in the country and by extension, enhance HRH planning.

## Competing interests

The author was a member of the technical secretariat of the High Level Expert Group on Universal Health Coverage, Planning Commission, and Government of India.

## Funding statement

None declared

